# Retraction: Kinetin mitigates Cd-induced damagesto growth, photosynthesis and PS II photochemistry of *Trigonella* seedlings by up-regulating ascorbate-glutathione cycle

**DOI:** 10.1371/journal.pone.0277593

**Published:** 2022-11-16

**Authors:** The PLOS ONE Editors

The *PLOS ONE* Editors retract this article [1] because it was identified as one of a series of submissions for which we have concerns about authorship, competing interests, and peer review. We regret that the issues were not addressed prior to the article’s publication.

GB, SS, and SMP did not agree with the retraction. MB responded but expressed neither agreement nor disagreement with the editorial decision. MJA, SU, SA, and SAA either did not respond directly or could not be reached.
